# Transcriptome response to temperature stress in the wolf spider *Pardosa pseudoannulata* (Araneae: Lycosidae)

**DOI:** 10.1002/ece3.2142

**Published:** 2016-04-20

**Authors:** Rong Xiao, Liang Wang, Yingshuai Cao, Guren Zhang

**Affiliations:** ^1^ State Key Laboratory for Biocontrol Sun Yat‐sen University Guangzhou China

**Keywords:** Gene regulation, *Pardosa pseudoannulata*, temperature stress, transcriptome, wolf spider

## Abstract

The wolf spider *Pardosa pseudoannulata* is a dominant predator in paddy ecosystem and an important biological control agent of rice pests. Temperature represents a primary factor influencing its biology and behavior, although the underlying molecular mechanisms remain unknown. To understand the response of *P. pseudoannulata* to temperature stress, we performed comparative transcriptome analyses of spider adults exposed to 10°C and 40°C for 12 h. We obtained 67,725 assembled unigenes, 21,765 of which were annotated in *P. pseudoannulata* transcriptome libraries, and identified 905 and 834 genes significantly up‐ or down‐regulated by temperature stress. Functional categorization revealed the differential regulation of transcription, signal transduction, and metabolism processes. Calcium signaling pathway and metabolic pathway involving respiratory chain components played important roles in adapting to low temperature, whereas at high temperature, oxidative phosphorylation and amino acid metabolism were critical. Differentially expressed ribosomal protein genes contributed to temperature stress adaptation, and heat shock genes were significantly up‐regulated. This study represents the first report of transcriptome identification related to the Araneae species in response to temperature stress. These results will greatly facilitate our understanding of the physiological and biochemical mechanisms of spiders in response to temperature stress.

## Introduction

**Figure 1:**
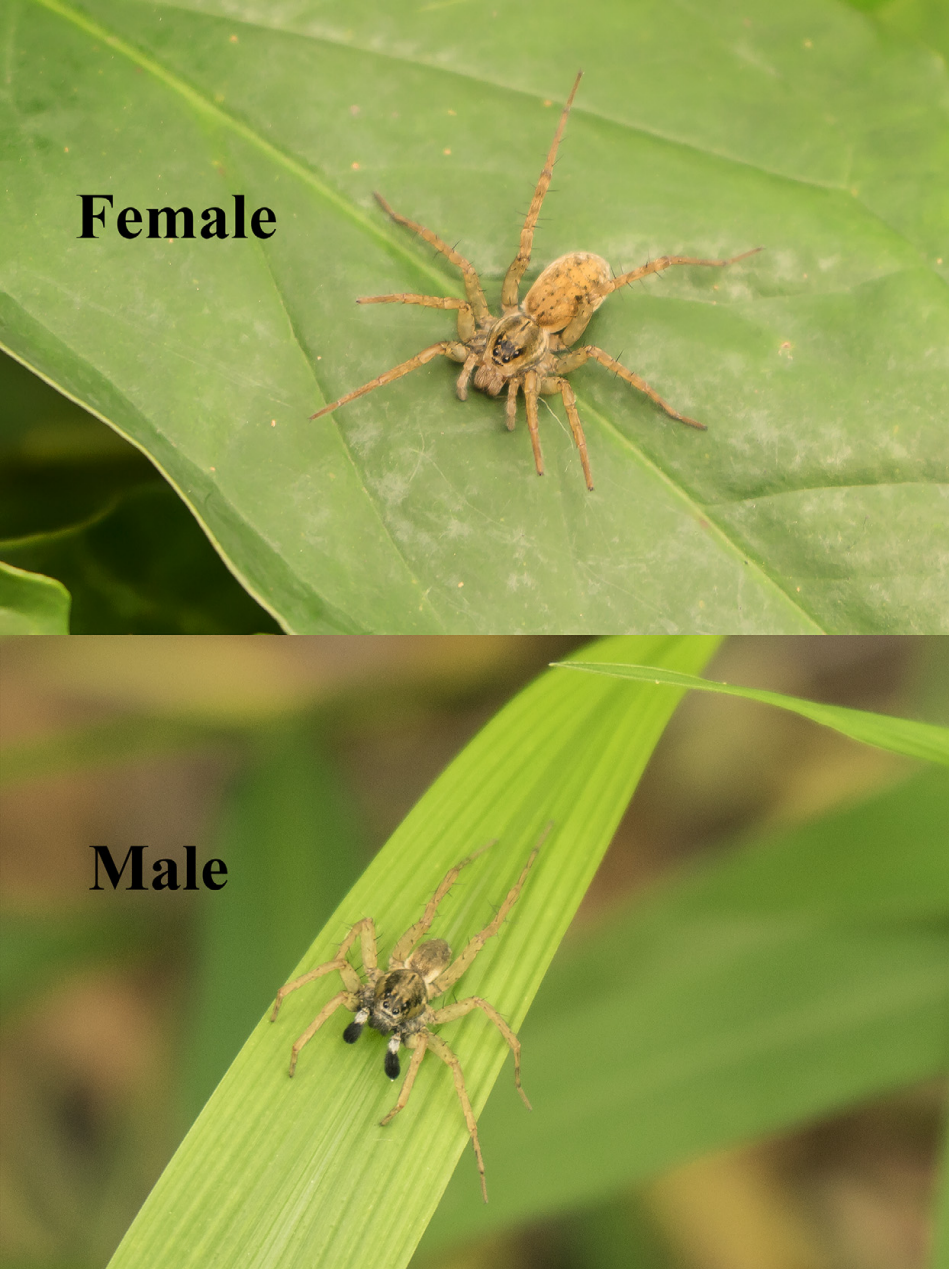
The female and male of *Pardosa pseudoannulata* in the field.

In the paddy ecosystem, spiders are important natural predator and represent a natural control factor for paddy insect pests (Barrion and Litsinger 1995; Haiming 1996). The wolf spider *Pardosa pseudoannulata* (Fig. 1) (Araneae: Lycosidae) is a dominant predatory species widely distributed in the rice ecosystem of the East Asia (World Spider Catalog 2016) and plays an important role in regulating the population densities of rice insect pests (Zhang et al. 1995; Zhao 2001; Li et al. 2002). Wolf spiders thus serve as effective biological control agents in the development and implementation of integrated pest management with rice ecosystems (Zhang et al. 1996).

The growth, development, survival, reproduction, and predatory behavior of *P. pseudoannulata* are significantly influenced by the ambient temperature in the rice ecosystems, which is often lower than 0°C in winter and higher than 40°C in summer (Sun et al. 2007). Comparatively, the optimal temperature of *P. pseudoannulata* is from 20°C to 30°C, whereas the developmental threshold temperature of males and females are 10.49°C and 9.75°C respectively. For this species, the maximum egg number and mean hatchability occur at 25°C, and the optimum temperature for development through the juvenile stages is at 30°C (Wang et al. 1982; Zhao et al. 1989; Li and Jackson 1996). In addition, temperature significantly influences the intervals of oviposition, the number of egg sacs, the survival rate of larvae, and the adult life‐span (Zhao et al. 1989). When the temperature is lower than 10°C, the spiders cease to feed, grow, and develop, and at temperatures higher than 40°C, the spiders act slowly, and most remain under objects or hide inside soil holes (Wang et al. 1982). How this spider species adapts to the stress of temperature variation and has become the dominant species in this ecosystem is unknown, and few references are available regarding how temperatures influence the physiology and biochemistry of *P. pseudoannulata* at the molecular level.

In recent years, the increasing use of high‐throughput sequencing has broadened our understanding of biological mechanisms of arachnids (Clarke et al. 2014; Haney et al. 2014). The magnitude of transcriptome response should be consistent with the change in whole animal performance, and it might be conjectured that spiders should exhibit minimal gene expression changes taking their optimal 25°C subsistence temperature as a balanced steady state (Windisch et al. 2014). In contrast, physiological response to thermal stress is manifold and complex, and many genes, proteins, metabolic pathways have been shown to be involved in this process in animals (Goto 2001; Overgaard et al. 2007; Teets et al. 2012; Zhao and Jones 2012; Wei et al. 2015). *P. pseudoannulata* might therefore possess a series of gene regulatory and metabolic response mechanisms to deal with thermal stress. It is therefore important to compare the transcriptome and expression profiling data for this species following exposure to extreme temperatures to obtain a better understanding of the biological mechanisms underlying its adaptation to extreme temperature.

The present study aimed to confirm the differential expression of genes in *P. pseudoannulata* in response to cold or hot stress, focusing on the identification of underlying regulatory metabolisms. Accordingly, adult spiders were exposed to 10°C, 25°C, and 40°C for 12 h and their respective transcriptomes were compared for the first time. The results of these transcriptome analyses might serve to build a framework for comprehensively understanding the biology and molecular mechanisms of *P. pseudoannulata* adaptation to thermal stress.

## Materials and Methods

### Biological materials and RNA extraction

Subadults of *P. pseudoannulata* were collected from the rice fields of South China Agricultural University, Guangzhou, Guangdong Province, China (113.37° E, 23.17° N). Spiders were reared individually in transparent glass tubes (2.5 × 10 cm, diameter × height) and fed in a climate box (PRX‐350C, Shanghai Baidian instrument limited company, China) at 25°C ± 0.5°C, relative humidity 60% ± 10% and 14L:10D. Spiders were supplied with water ad libitum and fed every 3 days with 4–6 adult *Musca domestica* houseflies. When spiders had matured for 2 weeks, individuals were transferred to man‐made climate boxes maintained at 10°C ± 0.5°C for the low temperature stress group (TL), or 40°C ± 0.5°C for the high temperature stress group (TH) for 12 h respectively. Spiders maintained at 25°C ± 0.5°C were used as controls (TC). Three biological replications were performed with each treatment containing three males and three females. The treated spiders were immediately frozen in liquid nitrogen and stored at −80°C refrigerator for RNA extraction.

The whole body (containing the carapace and abdomen) of each spider was used for RNA extraction, and then equal quality RNA of the three replicates of each group were mixed for mRNA‐sequencing. Total RNA was extracted from each sample using TRIzol reagent with standard protocols (Invitrogen, Waltham, MA) and then treated with DNase to remove any contaminating genomic DNA. RNA purity, concentration and integrity were evaluated using a Nanodrop (Thermo Fisher Scientific, Waltham, MA), Qubit 2.0 (Thermo Fisher Scientific), and Agilent 2100 (Agilent Technologies, Santa Clara, CA) respectively.

### mRNA library construction, sequencing, and de novo assembly

After confirming sample quality, three mRNA‐seq libraries (TC, TL, TH) were prepared for RNA sequencing using the NEB Next Ultra RNA Library Prep Kit for Illumina. In brief, magnetic beads coated with ploy (T) were used to enrich and purify the mRNAs from within the total RNA. Under a high temperature condition, fragmentation buffer was used to cut the mRNA into short fragments for selecting appropriately sized pieces. Using these fragments as templates, random hexamer primers were used to synthesize first‐strand cDNA. Then, mixture of buffer, dNTPs, RNase H, and DNA polymerase I were used to synthesize second‐strand cDNA. The cDNAs were purified and suitable fragments were selected using AMPure XP beads. Finally, cDNA libraries were obtained using PCR amplification. The resultant libraries were tested for quality conformance and sequenced using an Illumina HiSeq^™^ 2500 incorporating high‐throughput sequencing to generate 100 bp paired‐end read lengths.

Subsequently, the sequencing adaptor and primer sequences of the raw reads were discarded and low‐quality data were filtered to obtain clean reads (clean data). Short fragments (K‐mers) were obtained by applying Trinity software (Grabherr et al. 2011) to cleave clean reads. The K‐mers were configured into long segments (contigs), and then the overlap between these contigs was utilized to obtain fragment collections (components). Finally, using the method of the De Bruijn graph, the clean read information was utilized to obtain transcript sequences. After using TIGR gene indices clustering tools, the longest transcript of each group was chosen as the unigene; collectively, these constituted the unigene library.

### Unigene functional annotation

Unigene annotation information was obtained using BLASTx to align unigene sequences to NR (NCBI, nonredundant protein database), Swiss‐Prot, Gene Ontology (GO), Clusters of Orthologous Groups (COG), and Kyoto Encyclopedia of Genes and Genomes (KEGG) protein information databases. The parameter *E*‐value ≤10^−5^ of BLASTx was taken as the standard.

### Differential gene expression and functional annotation analyses

All clean read were aligned to unigene library using Bowtie software (Langmead et al. 2009). These results were employed to estimate expression level using RSEM software (Li and Dewey 2011). Fragments Per Kilobase of transcript per Million (FPKM) mapped reads were used to indicate the expression abundance of respective unigene (Trapnell et al. 2010). FPKM could eliminate the influences that gene length and sequencing quantity difference for calculation gene expression, the gene expression results could be directly used to compare the gene expression differences between different samples.

Because our sequence samples did not have biological replicates, we used EBSeq software (Leng et al. 2013) for differentially expressed gene (DEG) analysis. In the analysis procedure, we used recognized effective approaches such as the Benjamini‐Hochberg method to correct the significance of the *P*‐value (Benjamini and Hochberg 1995). To reduce the false positives among the expression values that occur when a large number of genes are analyzed by successive independent statistical analyses, we used corrected *P*‐values that called false discovery rate (FDR) as the key indicator for screening DEGs. In the process of screening, FDR <0.01 and log_2_fold change (FC) ≥1 were taken as the selection criteria.

Based on the gene expression levels of different samples, functional annotations for the DEGs were analyzed. Then, GO annotation statistical analysis of DEGs in “biological process” (BP), “cellular component” (CC), “molecular function” (MF) three main categories between groups was implemented. GO enrichment analysis was used the topGO software (Alexa et al. 2006) by the “elim” method with a minimum node size of 6.

On the basis of GOG and KEGG annotation, we statistically analyzed the DEGs in COG function classifications and KEGG pathway classifications respectively. We further analyzed DEGs in the KEGG pathway enrichment degree by the enrichment factor and corrected *P*‐value (*Q*‐value). Computation formula of *Q*‐value is as follows, *Q*‐value = (the number of DEGs/the number of all unigenes in KEGG)/(the number of DEGs in pathway/the number of all unigenes in pathway). The enrichment factor is smaller, the more significant of the enrichment level for DEGs, and the log value of *Q*‐value is bigger, the more reliable of the enrichment significant for DEGs.

### Validation of mRNA‐seq data

To evaluate the quality of the mRNA‐seq data and expression level, six annotated unigenes were selected randomly and amplified using reverse transcripts (RT)‐PCR and were quantified by real time quantitative PCR (qRT‐PCR). Total RNA was extracted from the previous samples and conducted as described above. The primer sets for each unigene were designed using Primer Premier 5.0 software (Tables S1, S2). The cDNA of unigenes was synthesized according to the manufacturer's protocol (PrimeScriptRT reagent Kit; TaKaRa, Kusatsu, Shiga, Japan). The amplified products of RT‐PCR were cloned into pGEM‐T Easy Vector Systems (Promega, Madison, WI) after purified from the gel, then for Sanger sequencing (Invitrogen Biotechnology, Guangzhou, China). All the unigenes sequenced target fragments were aligned to the nonredundant transcripts in mRNA‐seq using BLASTn (E‐value ≤10^−5^).

qRT‐PCR was performed using LightCycler^®^ 480 SYBR‐Green I Master (Roche Diagnostics, Basel, Switzerland) and run on the LightCycler^®^ 480 Real‐time PCR system (Roche Diagnostics Ltd). Data for each sample were normalized in relation to the internal control gene (*β‐actin*) using the 2^−ΔΔCT^ method (Livak and Schmittgen 2001). All samples were tested in triplicate, and the experiments were performed on three biological replicates.

## Results

### mRNA sequencing, assembly, functional annotation

To study the mRNA expression dynamics of *P. pseudoannulata* under different thermal stresses, we constructed and sequenced mRNA‐seq libraries from adults reared at 25°C as a neutral control (TC), or exposed to low (10°C, TL) and high (40°C, TH) temperature stresses respectively. After controlling for the quality of sequencing data, we obtained a total of 10.3 Gb clean data, with Q30 percentages (A Q‐score of 30 corresponds to an incorrect base rate of 1 per 1000) of each sample not less than 90.85% from the three mRNA‐seq libraries (Table 1). The reliability of the sequencing quality is positively correlated with the Q30 ratio. In general, the quality of the sequencing is very reliable when the Q30 percentages ≥80%. De novo assembly of the clean reads was then performed and 96,707 transcripts representing 67,725 unigenes were obtained. The N50s of the transcripts and unigenes were 1735 and 1308 respectively, and 13,762 unigenes were over 1 kb (Table 2). Usually, the completeness of the assembly was good as the N50s of the unigenes ≥800 bp.

**Table 1:** Data analysis of clean reads from *Pardosa pseudoannulata* mapped to the reference transcriptome

Sample name	TC	TL	TH
Raw reads number	19,078,944	16,423,026	15,980,253
Clean reads number	19,014,805	16,240,779	15,911,017
Total mapped reads	16,136,606 (84.86%)	13,237,006 (81.50%)	13,223,450 (83.11%)
Uniquely mapped reads	12,995,710 (80.54%)	10,558,070 (79.76%)	10,524,303 (79.59%)
Multiple mapped reads	3,140,896 (19.46%)	2,678,936 (20.24%)	2,699,147 (20.41%)
Bases number	3,840,510,457	3,279,979,674	3,213,524,760
GC content	43.54%	44.23%	44.40%
Q30	90.86%	91.10%	91.86%

**Table 2:** Data analysis of *Pardosa pseudoannulata* clean reads in assembly results

Length range	Contig	Transcript	Unigene
0–300	12,891,044 (99.63%)	28981 (29.97%)	23753 (35.07%)
300–500	19454 (0.15%)	22933 (23.71%)	17196 (25.39%)
500–1000	14275 (0.11%)	19843 (20.52%)	13013 (19.21%)
1000–2000	8559 (0.07%)	13903 (14.38%)	8378 (12.37%)
2000+	5374 (0.04%)	11045 (11.42%)	5384 (7.95%)
Total Number	12,938,706	96,707	67,725
Total Length	531,081,896	89,746,362	51,053,313
N50 Length	42	1735	1308
Mean Length	41.05	928.02	753.83

**Figure 2:**
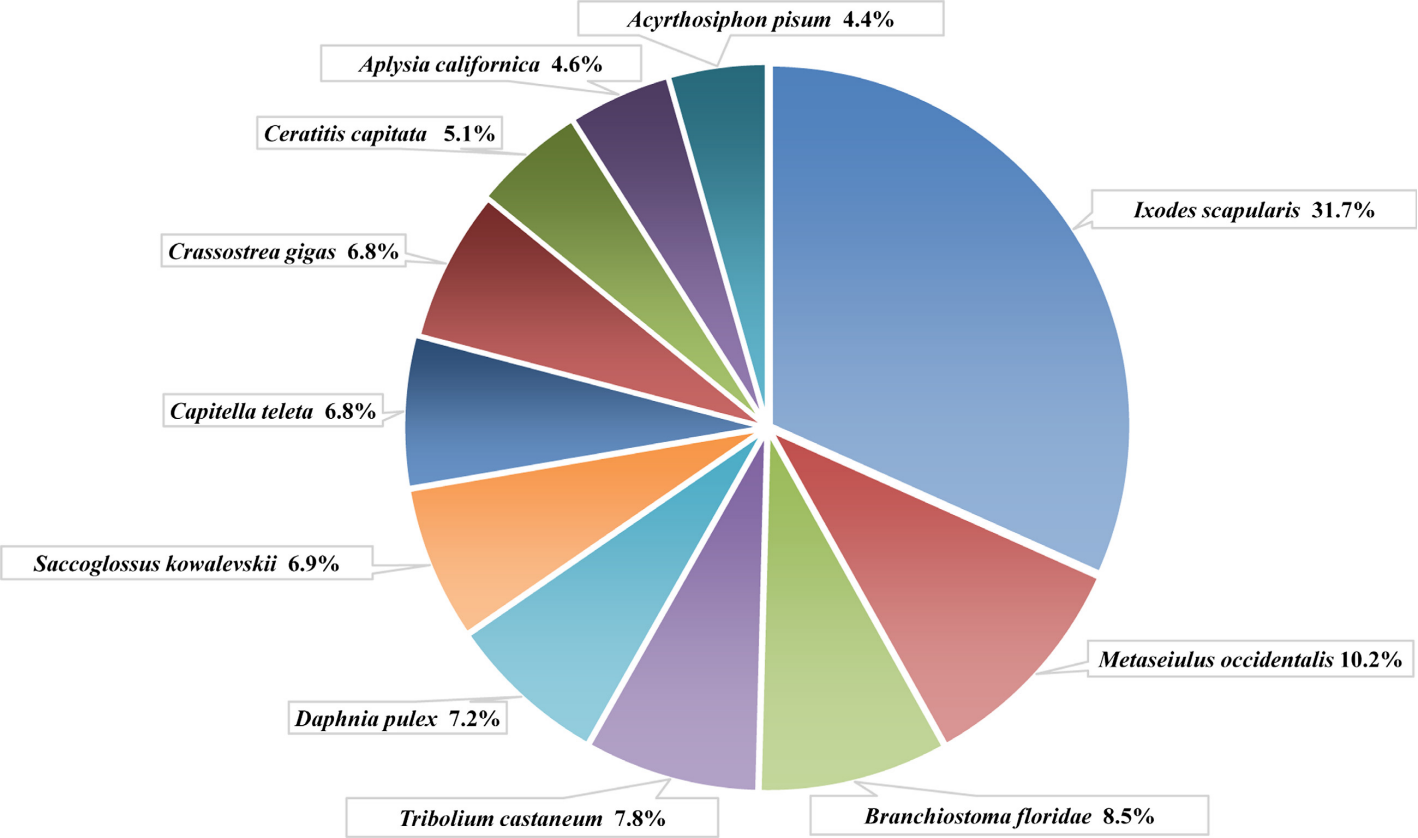
Species distribution of the BLASTx results based on the NR database for all annotated unigenes.

Functional annotation was subsequently performed using NR, Swiss‐Prot, KEGG, COG, and GO databases and a total of 21,765 unigene annotations were finally obtained. Based on the NR annotation analysis, the species distribution of the best match result for each unigene is shown in Figure 2. There were 3760 (31.7%) unigene sequences that preferentially matched with the species *Ixodes scapularis*, and 1206 (10.2%) unigene sequences matched with the species *Metaseiulus occidentalis*. Araneae were a genome resource poor arthropod order, so the annotated unigenes were lesser.

### Differential gene expression

**Figure 3:**
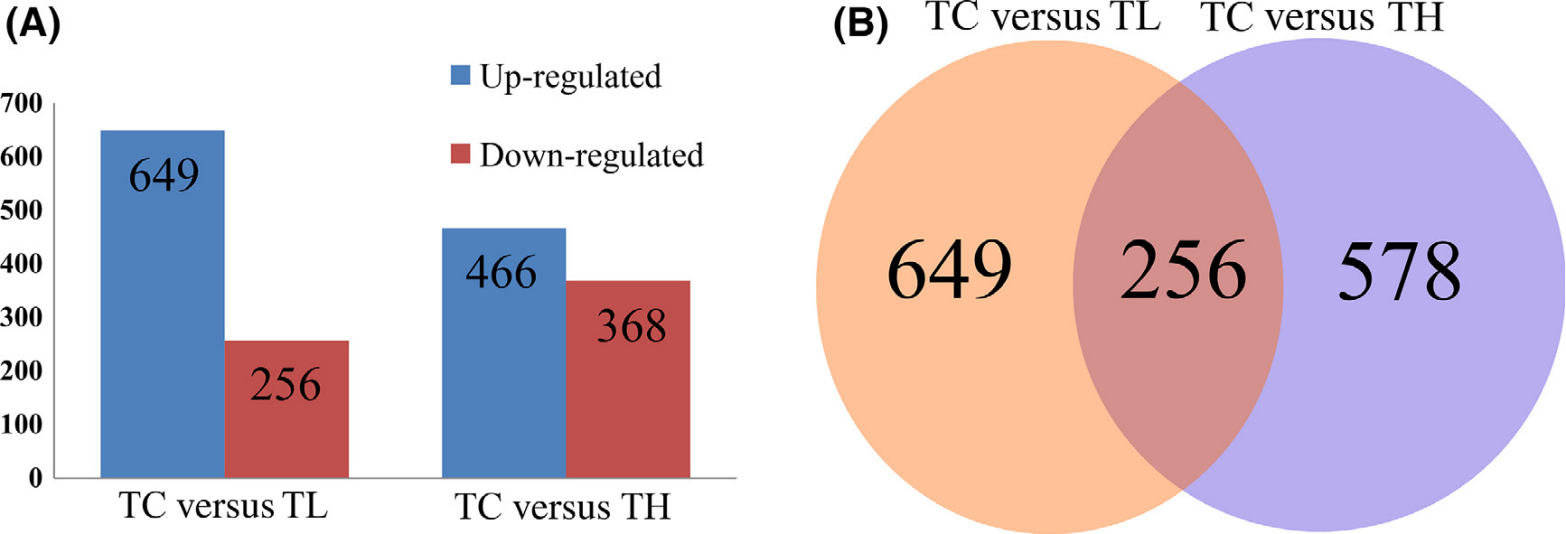
The differentially expressed genes numbers associated with low (TC vs. TL) and high (TC vs. TH) temperature stress.

A total of 905 (649 up‐ and 256 down‐regulated) and 834 (466 up‐ and 368 down‐ regulated) responsive genes from low and high temperature stress were identified, respectively, with an FDR <0.01 and log_2_FC ≥1 taken as the selection criteria (Fig. 3A). The extent of up‐ and down‐regulation was similar under low and high temperature stresses. Through functional annotation, we separately obtained 692 and 602 annotated DEGs in TC versus TL and TC versus TH respectively. Notably, 256 genes were commonly regulated by low and high temperature stress (Fig. 3B). Among, nine of these were down‐regulated in TL but up‐regulated in TH, among which only six genes were annotated, albeit for a hypothetical protein. On the other hand, three genes were up‐regulated in TL but down‐regulated in TH, only one of which was also annotated for hypothetical protein. In summary, we might speculate all of these DEGs including the seven hypothetical proteins might be involved in the thermal adaptation of *P. pseudoannulata*.

A total of 106 and 41 differentially expressed mitochondrial ribosomal genes from low and high temperature exposure were, respectively, annotated into the KEGG ribosome pathway and these genes as a group were suggested to participate in translation, ribosomal structure, and biogenesis (COG, J). Under low temperature stress, there were 73 up‐regulated and 33 down‐regulated ribosomal genes. Under high temperature stress, all 41 ribosomal genes were down‐regulated; 22 ribosomal genes were simultaneously identified as being down‐regulated at both low and high temperature.

We identified four and nine differentially expressed heat shock protein (HSP) genes as showing up‐regulated expression patterns when the spiders, respectively, endured low and high temperature stresses (Table 3), all of which were classified in posttranslational modification, protein turnover, chaperones (COG, O) categories. Among these HSPs, two genes were classified to the Hsp70 family and were commonly up‐regulated during both low and high temperature stresses. Also c52211.graph_c0 and c58352.graph_c0 were categorized to the Hsp70 family as well and were up‐regulated only under high temperature stress. In addition, two genes belonged to the superfamily of small heat‐shock proteins, two genes belonged to the Hsp60 superfamily, and another three genes belonged to the Hsp90 superfamily (Table 3).

**Table 3:** Differentially expressed heat shock protein (HSP) genes in *Pardosa pseudoannulata* during temperature stresses

	Gene‐ID	Gene length(bp)	TC	TL(TH)	FDR	Regulated	Gene description
TC versus TL	c44708.graph_c0	975	349.4503	693.5493	1.26E‐05	Up	Hsp27
	c52721.graph_c0	2818	10.31822	31.69584	7.77E‐15	Up	Hsp70B2
	c12445.graph_c0	2531	0	10.09792	0	Up	Hsp83
	c56853.graph_c0	2318	0	14.56156	0	Up	Hsp70
TC versus TH	c37924.graph_c0	2110	50.63419	120.1325	6.58E‐06	Up	Hsp60
	c33263.graph_c0	785	50.81281	133.4898	1.59E‐07	Up	Hsp10
	c52211.graph_c0	2422	67.97175	198.0208	1.60E‐09	Up	Hsp70B2
	c53702.graph_c0	2456	155.4408	467.6965	6.16E‐10	Up	Hsp83
	c58924.graph_c0	538	0.125786	3.530414	9.50E‐05	Up	Hsp60
	c58352.graph_c0	459	0.149172	4.36884	4.98E‐05	Up	Hsp70
	c56853.graph_c0	2318	0	0.57567	0.000584	Up	Hsp70
	c52721.graph_c0	2839	10.31876	356.9711	0	Up	Hsp70B2
	c54483.graph_c0	3451	36.31718	93.5473	2.85E‐07	Up	Hsp90

### Functional enrichment analysis of DEGs

**Figure 4:**
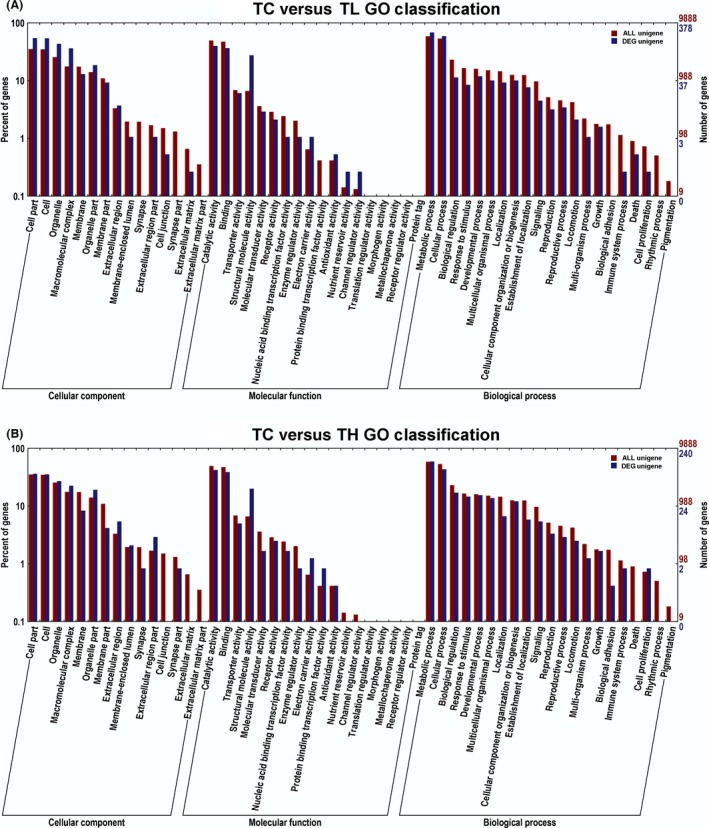
Histogram of Gene Ontology (GO) classification of differentially expressed genes (DEGs) in TC versus TL (A) and TC versus TH (B). The red histogram indicates all the annotated uigenes in each subcategory, the blue histogram indicates all the annotated DEGs in each subcategory. The right *y*‐axis indicates the number of annotated genes in each subcategory, the red numbers corresponding to the annotated uigenes, the blue numbers corresponding to the annotated DEGs. The left *y*‐axis indicates the percentage of annotated uigenes or DEGs in that main category.

The functional classification of DEGs was further analyzed to explore the pattern of transcriptome regulation that occurs during low and high temperature stress. First, we performed GO enrichment analysis of DEGs that were identified between the groups (TC vs. TL and TC vs. TH) among the three main clusters respectively (Fig. 4). Forty‐one and forty‐two subcategories under three main GO categories were obtained when spiders were exposed to low and high temperatures respectively. The predominant enriched subcategories both in TC versus TL and TC versus TH were shown in Table 4.

**Table 4:** The predominant enriched subcategories both in the groups TC versus TL and TC versus TH

Cellular component	Molecular function	Biological process
“cell” GO:0005623	“catalytic activity” GO:0003824	“metabolic process” GO:0008152
“cell part” GO:0044464	“binding” GO:0005488	“cellular process” GO:0009987
“macromolecular complex” GO:0032991	“structural molecule” GO:0005198	“biological regulation” GO:0065007
“organelle” GO:0043226	“electron carrier activity” GO: 0009055	“cellular component organization” GO:0016043
“organelle part” GO:0044422	“antioxidant activity” GO:0016209	“cellular component biogenesis” GO:0044085

TopGO was further used for the enriched GO terms in the three primary clusters (enrichment significance KS <0.05). The subcategories that topGO significant enrichment GO terms belong to were consistent with the predominant enriched subcategories that shown in Table 4. Detailed information of the topGO enrichment of the groups TC versus TL and TC versus TH were shown in Table S3. However, transporter activity (part of MF) and transferase activity, transferring glycosyl groups (part of MF) were significantly enriched GO terms in TC versus TL but not in TC versus TH. Because when spiders under low temperature stress, they need more energy. Therefore, these DEGs played a significant role when the spiders suffered from low temperature stress.

**Figure 5:**
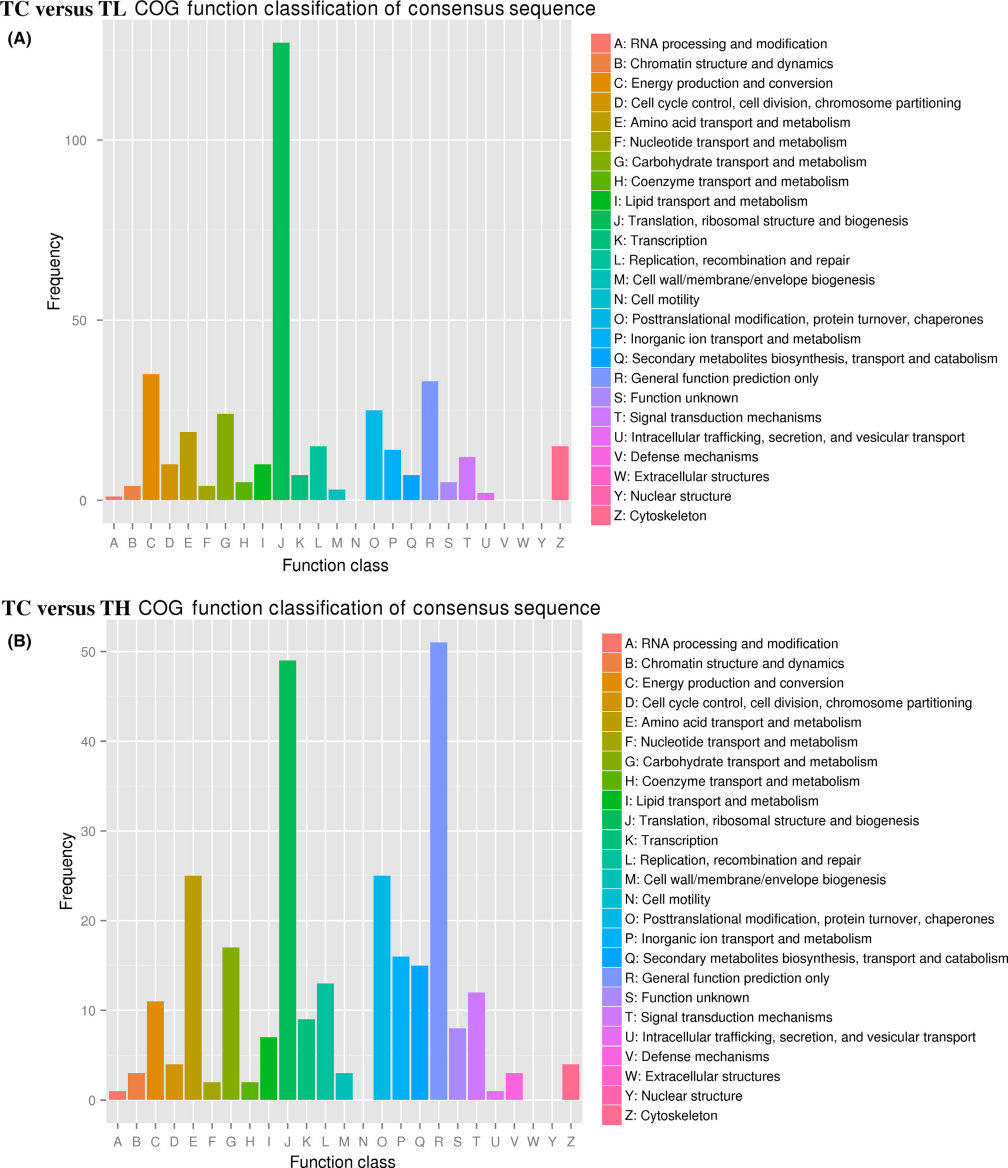
Clusters of Orthologous Groups consensus sequence functional classification of differentially expressed genes in TC versus TL (A) and TC versus TH (B).

The DEG annotation results in 26 COG classifications of TC versus TL and TC versus TH are shown in Figure 5. In TC versus TL, the DEGs were mainly localized into the following four classifications: J (33.69%), C (9.28%), O (6.63%), and G (6.36%) (Fig. 5A). On the other hand, in TC versus TH, J (17.44%), E (8.90%), O (8.90%), and G (6.05%) were the four primary classifications (Fig. 5B). So, energy production and conversion play an important role when spiders under low temperature, while amino acid transport and metabolism play a vital role under high temperature.

**Figure 6:**
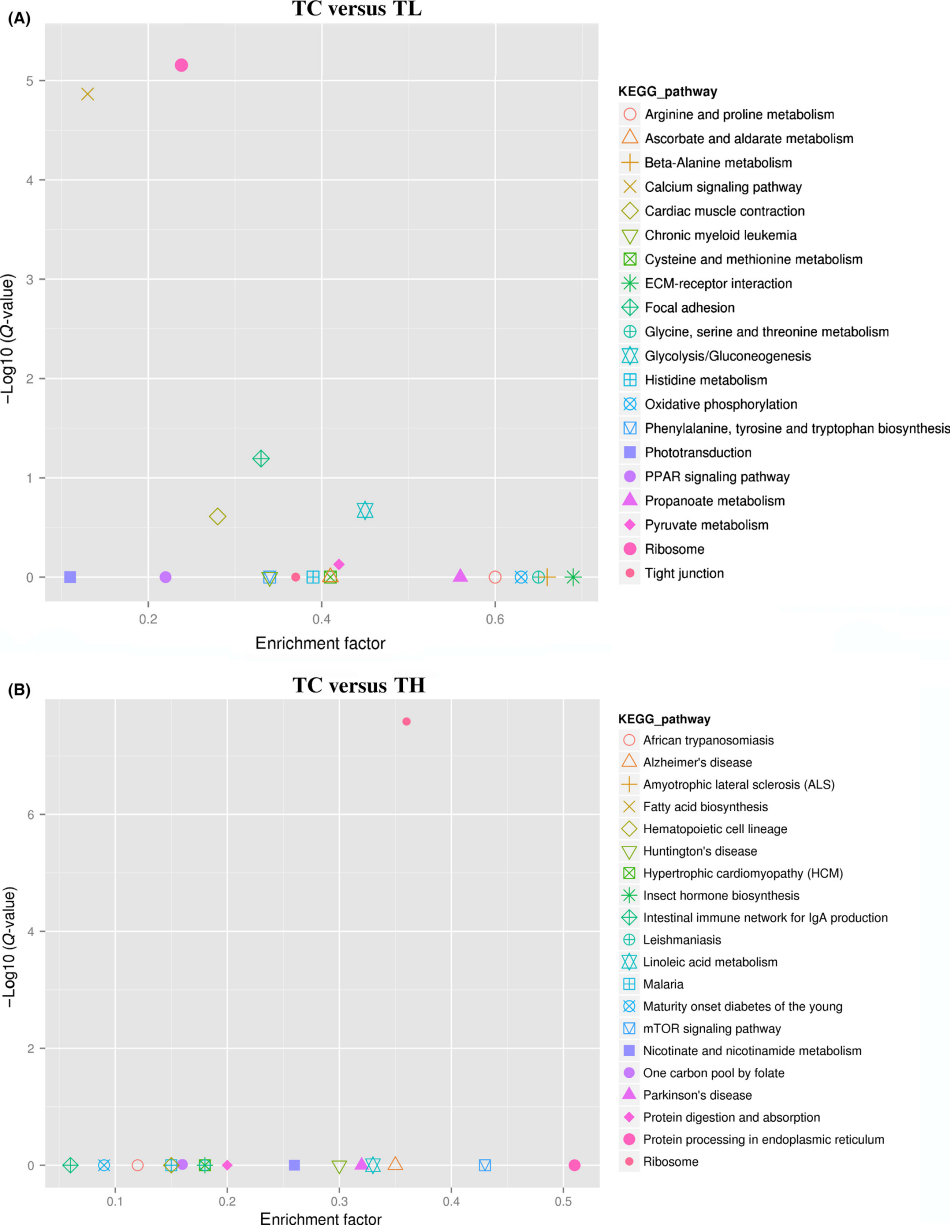
Kyoto Encyclopedia of Genes and Genomes pathways Enrichment analysis of differentially expressed genes in TC versus TL (A) and TC versus TH (B).

The DEGs annotation results from KEGG pathways type classification showed that 87 and 84 pathways were differentially expressed in TC versus TL and TC versus TH respectively. We then analyzed the significant of the pathways using an enrichment factor and the *Q*‐value; the results of the first 20 minimum *Q*‐value pathways are displayed in Figure 6. In the group TC versus TL and TC versus TH, the dominant and significantly enriched pathways were shown in Table 5. Under high temperature stress, protein processing in endoplasmic reticulum, spliceosome, lysosome were also significant enriched pathways.

**Table 5:** The dominant and significantly enriched pathways in the groups TC versus TL and TC versus TH

TC versus TL	TC versus TH
Ribosome (106 DEGs, 43.8%)	Ribosome (41 DEGs, 31.3%)
Oxidative phosphorylation (25 DEGs, 10.3%)	Protein processing in endoplasmic reticulum (12 DEGs, 9.2%)
Glycolysis/gluconeogenesis (15 DEGs, 6.2%)	Spliceosome (5 DEGs, 3.8%)
Focal adhesion (11 DEGs, 4.5%)	Lysosome (4 DEGs, 3.1%)
Calcium signaling pathway (10 DEGs, 4.1%)	Oxidative phosphorylation (4 DEGs, 3.1%)
Pyruvate metabolism (10 DEGs, 4.1%)	Purine metabolism (4 DEGs, 3.1%)
Arginine and proline metabolism (9 DEGs, 3.7%)	Arginine and proline metabolism (3 DEGs, 2.3%)

At low temperature, the involved DEGs in the oxidative phosphorylation pathway were all up‐regulated and participated in energy production and conversion (COG, C). We found that 12 key genes were annotated for different subunits of reduced nicotinamide adenine dinucleotide (NADH) dehydrogenase. Five additional key genes were annotated for different cytochrome oxidase subunits, and five were annotated for different ATP synthase subunits. In the pathway of pyruvate metabolism pathway, two key genes were annotated for pyruvate kinase and participated in carbohydrate transport and metabolism (COG, G). A further eight key genes participated in energy production and conversion (COG, C); among them, three genes were annotated for aldehyde dehydrogenase, two were l‐lactate dehydrogenases, two were malate dehydrogenases, and the remainder was acylphosphatase. In the glycolysis/gluconeogenesis pathway, 15 key genes were identified, seven of which were as those annotated for pyruvate kinase, aldehyde dehydrogenase, and l‐lactate dehydrogenase in the pyruvate metabolism pathway. The other eight key genes all participated in carbohydrate transport and metabolism (COG, G): glyceraldehyde‐3‐phosphate dehydrogenase (3), fructose‐bisphosphate aldolase (2), enolase (1), 6‐phosphofructokinase (1), and phosphoglycerate mutase (1).

At high temperature, oxidative phosphorylation (four DEGs, 3.1%), purine metabolism (four DEGs, 3.1%), arginine and proline metabolism (three DEGs, 2.3%), cysteine and methionine metabolism (three DEGs, 2.3%), amino sugar and nucleotide sugar metabolism (three DEGs, 2.3%), one carbon pool by folate (three DEGs, 2.3%), and drug metabolism‐cytochrome P450 (three DEGs, 2.3%) were the dominant metabolic pathways. In the pathway of oxidative phosphorylation, all four involved DEGs were up‐regulated, and they all participated in energy production and conversion (COG, C); only one key gene was annotated as a subunit of NADH dehydrogenase. The involved key DEGs in cysteine and methionine and arginine and proline metabolic pathways were down‐regulated, and participated in amino acid transport and metabolism (COG, E).

### Validation of mRNA‐seq data

We selected six annotated DEGs (c50430.graph_c0, c43733.graph_c0, c27804.graph_c0, c34593.graph_c0, c47921.graph_c0, c21981.graph_c0) for RT‐PCR validation. In this analysis, all six primer pairs for the respective unigenes yielded bands with the expected size and the identities of the PCR products were confirmed by Sanger sequencing (Table S1). The exact identities between the RT‐PCR fragments and the unigenes from mRNA‐Seq (BLASTn, E‐value ≤10^−5^) indicated the reliability of our transcriptome data.

**Figure 7:**
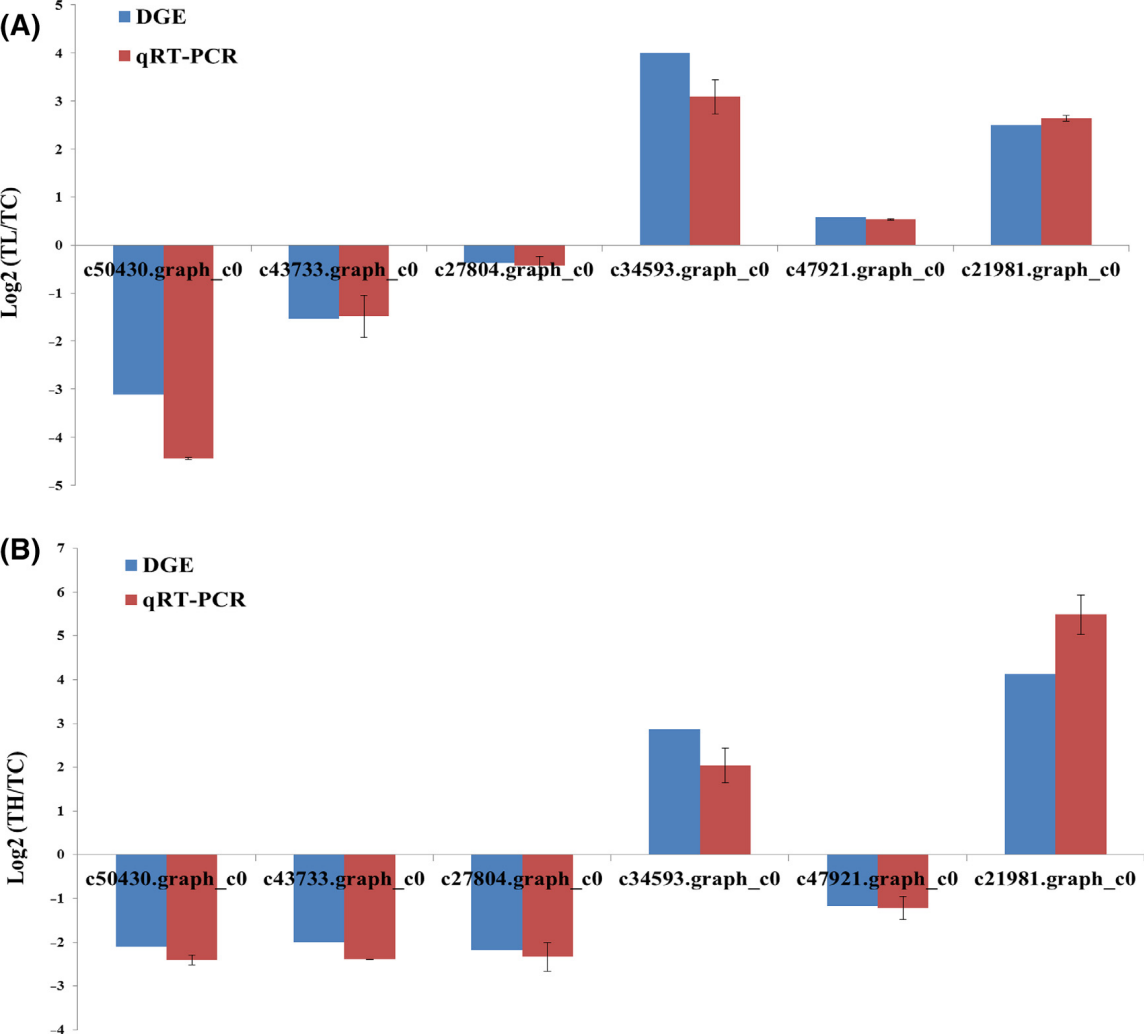
qRT‐PCR validation of mRNA‐seq data for unigene expression levels. The *y*‐axis indicates the relative expression levels, (A) represent each unigene in TL compared with TC, (B) represent each unigene in TH compared with TC, each error bar indicates the standard error.

To confirm the DEG results obtained by Illumina sequencing, we performed qRT‐PCR for the unigenes validated above. Data are presented as fold changes in expression normalized to the *β‐actin* and relative to the TC sample (Fig. 7). Although the fold changes of some unigenes were not completely consistent, possible due to different methods of library construction and so on, these results indicated the reliability of the differential expression results.

## Discussion

### mRNA Sequencing, assembly, functional annotation

Using Illumina deep sequencing of mRNAs expressed in three temperature treatments, we generated a high quality transcriptome for the *P. pseudoannulata*. We obtained more than 10.3 GB clean reads that were assembled into 96,707 transcript and 67,725 unigenes, with a mean length of 928 bp and 753 bp respectively. After assembly, 53.68% transcripts and 60.46% unigenes were still less than 500 bp, which might be due to the short length sequencing capacity of illumine sequencing. To date, *P. pseudoannulata* do not have genomic resource, transcriptome research also is less, only adult spider *P. pseudoannulata* cephalothoraxes transcriptome have been analyzed for genes related to insecticide effects and detoxification (Meng et al. 2015). Our transcriptome NR annotation analysis results were in accordance with theirs, that *I. scapularis* and *M. occidentalis* were the two top species matched to the transcriptomes (Meng et al. 2015). The unigene sequences primarily matched with Ixodida and Parasiformes, as no reference genome has previously reported in Lycosidae. The unigenes annotation results showed that only 21,765 (32.14%) uigenes had significant match in any of the existing databases, mainly because the lacking for the genomic and transcriptome information for the spiders currently.

### Differential gene expression

Through comprehensively investigate the differences in gene expression between samples, we obtained the large number of DEGs annotated for ribosomal components, the most substantial impact occurred in the translation process when spiders suffered from thermal stress (Figs. 5, 6). High mitochondrial densities have been found in other cold‐acclimated poikilotherm species such as fish (Clarke and Johnston 1999; Windisch et al. 2014), with a positive impact demonstrated wherein mitochondrial ribosomal proteins displayed high transcription rates at low temperatures (Windisch et al. 2014). The protein synthesis machinery is one of the largest multienzyme complexes in cells and, ribosome formation and its functioning were concerned with the majority of universal proteins (Korobeinikova et al. 2012). Ribosomal proteins were less expressed in *P. pseudoannulata* exposed to high temperature. Although we did not measure protein biosynthesis, it is likely that in the spider, these genes contributed to assist with protein biosynthesis compensates for protein damage in the adaption to thermal stress.

Heat shock proteins are a group of chaperone proteins that exist ubiquitously from prokaryotes to eukaryotes. HSPs maintain cell survival and a stable internal environment, and participate in the cellular responses to a variety of physiologically relevant conditions (Lemaux et al. 1978; Kelley and Schlesinger 1982; Kregel 2002; Chang 2009). In spite of little difference in the main functions among the different families, HSPs have been suggested to act as auxiliary proteins to assist with correct folding, assembly and disassembly, and transport and positioning between organelles (Lindquist and Craig 1988; Parsell and Lindquist 1993; Young et al. 2004). Under both low (Martinez et al. 2001; Sonna et al. 2002) and high temperature stress (Zhao et al. 2009, 2010), HSPs are rapidly and intensely induced in animals. Hsp70 and hsp90 are two typical HSPs widely documented to fulfill the role of cold shock chaperones in *Escherichia coli* (Lelivelt and Kawula 1995), *Trichinella spiralis* larvae (Martinez et al. 2001), and Quahog Parasite Unknown (Lelivelt and Kawula 1995; Rubin et al. 2014). In *P. pseudoannulata*, Hsp70 and hsp90 were up‐regulated following both low and high temperature, consistent with the finding that hsp70 and hsp90 were up‐regulated in *P. astrigera* by temperatures higher or lower than normal (Li et al. 2015). All up‐regulated HSPs under both low and high temperature stress were widely believed to function as turnover and chaperone proteins participating in endocytosis (ko04144), spliceosome (ko03040), and protein processing in the endoplasmic reticulum (ko04141), resulting in the adaption to temperature stresses. The question of how the HSPs of *P. pseudoannulata* perform their functions is a worthy topic for further investigation.

Noteworthy, although the significant enrichment GO terms and COG classification of most DEGs annotated in TC versus TL and TC versus TH were similar, their detailed annotated information differed. This suggested that the final functions of these DEGs were similar in TC versus TL and TC versus TH, and that they played an important role when the spiders suffered from temperature stress in either direction. If we want to study of these functions in depth in future, we should begin from these DEGs.

### Signal transduction

Cold‐specific signal transduction mechanisms are mainly comprised of calcium signaling pathway (Fig. 6A). Numerous studies have demonstrated that Ca^2+^ plays a vital role in the cold‐stress response of plants, for example in *Arabidopsis* (Knight et al. 1996; Polisensky and Braam 1996; Jakoby et al. 2002), banana (Yang et al. 2015), and rice (Saijo et al. 2000; Ma et al. 2015). The first report about the importance of Ca^2+^ as an important second messenger in animals was from the rapid cold‐hardening response in larvae of the Antarctic midge *Belgica antarctica* (Teets et al. 2008). Our study showed that most of the DEGs involving in signal transduction when spiders suffered from low temperature stress were also related to calcium signaling pathways, including orthologs of the calcium‐transporting ATPase sarcoplasmic/endoplasmic reticulum (five genes), ADP, ATP carrier protein (two genes), and voltage‐dependent anion‐selective channel protein (two genes). All nine of these genes in *P. pseudoannulata* were up‐regulated in response to low temperature stress and all participated in inorganic ion transport and metabolism (COG, P). In addition, a key gene (troponin C, TnC) of the calcium signaling pathway, was significantly up‐regulated and mainly participated in signal transduction mechanisms (COG, T). These results suggest that the calcium signaling pathway of *P. pseudoannulata* might quickly and effectively regulate downstream signaling and gene expression in response to low temperature stress.

Under high temperature, the mTOR signaling pathway was significant identified, and its key genes serine/threonine‐protein kinase and GTPase‐activating protein were down‐regulated in response to nutrient and cellular energy status (Inoki et al. 2003). In addition, the TGF‐beta signaling pathway also was identified in high temperature stress, this pathway has an important regulatory role in development and in extracellular matrix synthesis and would repair (Kingsley 1994), therefore, it might contribute to reduce the level of damaged proteins when spiders are under high temperature stress.

### Metabolism

For ectothermic animals various temperature ranges are thought to be associated with complicated systemic states involving alternative enzyme and metabolic patterns (Wieser 1973). Energy production and conversion is essential when poikilotherms suffered from cold temperature stress. Oxidative phosphorylation (25 DEGs, 10.3%), glycolysis/gluconeogenesis (15 DEGs, 6.2%), pyruvate metabolism (10 DEGs, 4.1%) and, arginine and proline metabolism (nine DEGs, 3.7%) were the primary metabolic pathways identified when spiders suffered from low temperature stress. Under aerobic conditions, glucose breaks down into pyruvate and enters the triglyceride acid cycle through oxidative phosphorylation synthesis of ATP. Under anaerobic conditions, glucose breaks down first into pyruvate, and then through glycolysis/gluconeogenesis synthesis of ATP (Zhang 2003). NADH, cytochrome, and ATP synthase (key genes) participate in electron transport as respiratory chain components, and have close relationships with the mitochondrial membrane in the process of energy release (Zhang 2003). These results indicated that the function of mitochondria was enhanced to release energy when spiders were under cold stress. Therefore, oxidative phosphorylation, glycolysis/gluconeogenesis and pyruvate metabolism all play important roles in ATP synthesis and provision energy when spiders suffer from low temperature stress.

Insects have evolved a suite of physiological strategies to cope with environmental temperature variation such as metabolic cold adaptation (Clarke 1993; Addo‐Bediako et al. 2002). A significant negative relationship was found between mean annual temperature and metabolic rate at the interspecific level, with lower environmental temperatures correlating with higher metabolic rates (Addo‐Bediako et al. 2002). Although we did not test the metabolic rate in spiders, the DEGs were shown to play important roles in metabolic function when spiders suffered from cold stress.

Our results indicated that the metabolism of spiders in the cold was stronger than that at high temperature. In aquatic ectotherms such as mussels, extreme warming temperatures would result in a reduction in aerobic scope or induce metabolic depression (Pörtner 2001, 2010; Múgica et al. 2015). The elevation of energy demand during thermal stress is commonly associated with reallocation of energy to support maintenance costs, requiring less energy to be allocated to growth, storage, and reproduction (Kooijman 2010; Múgica et al. 2015). When spiders remain at 10°C, the critical temperature for activity, they would be expected to produce more energy to maintain survival. In contrast, spiders under 40°C could act and reproduce normally, and spend more energy on maintaining growth, development, and reproduction. Accordingly, to trade off for the use of energy, the spiders would have to maintain an active metabolism at 10°C and produce less energy at 40°C relative to that at 25°C.

## Conclusions

In summary, we described the analysis of mRNA libraries generated from the wolf spider species of *P. pseudoannulata* following thermal stress in this study which was the first monitoring of temperature‐conditioned transcriptomes of *P. pseudoannulata*, as well as being the first to address the gene expression levels associated with temperature‐dependent physiological performance as a result of acclimation. We identified 905 and 834 genes exhibiting differential expression in response to low and high temperature stress in *P. pseudoannulata* respectively. Calcium signaling pathway played a vital role in the cold‐stress response of *P. pseudoannulata*. DEGs expressed in *P. pseudoannulata* were enriched for functions primarily involved energy production and conversion at low temperatures such as oxidative phosphorylation, glycolysis/gluconeogenesis and pyruvate metabolism, whereas DEGs showed enrichment for amino acid transport and metabolism following high temperature. HSP genes annotated from DEGs as primarily participating in posttranslational modification, protein turnover, chaperones were differentially expressed when spiders suffered from low and high temperature stress.

## Conflict of Interest

The authors have declared that they have no competing interests.

## Data Accessibility

Transcriptome sequencing raw data are available in the NCBI Short Archive (SRA) database with accession number SRX1244902, including the transcriptome TC (SRR2404407), transcriptome TL (SRR2405992), transcriptome TH (SRR2406000).

## Supporting information


**Table S1.** Primers used to validate the mRNA ‐seq data for RT‐PCR.
**Table S2.** Primers used to validate the mRNA ‐seq data for qRT‐PCR.
**Table S3.** Detailed information of topGO enrichment of the groups TC versus TL (low temperature) and TC versus TH (high temperature) and their subcategories.
