# Supplementary material for: Transcriptome response to temperature stress in the wolf spider Pardosa pseudoannulata (Araneae: Lycosidae)
Source: Ecol Evol. 2016 Apr 20;6(11):3540–54. doi: 10.1002/ece3.2142 (PMC4842027; doi:10.1002/ece3.2142)
Supplement: Supplementary file 1 — Table S1. Primers used to validate the mRNA ‐seq data for RT‐PCR. Table S2. Primers used to validate the mRNA ‐seq data for qRT‐PCR. Table S3. Detailed information of topGO enrichment of the groups TC versus TL (low temperature) and TC versus TH (high temperature) and their subcategories. [file ECE3-6-3540-s001.docx]

**Table S1.** Primers used to validate the mRNA -seq data for RT-PCR

| the primers for RT-PCR(5'-3') | | length | Identities | Gaps | E-value |
| --- | --- | --- | --- | --- | --- |
| *β-actin*F | GACCCAATACTTCTAACG | 1249bp | 100% | 0% | 0 |
| *β-actin*R | ACAGCAGGAAACACTTA |  |  |  |  |
| c50430.graph_c0-F | TCCAAGGCAGTGATACG | 780bp | 100% | 0% | 0 |
| c50430.graph_c0-R | CAAGGAGGACAAGGACC |  |  |  |  |
| c43733.graph_c0-F | AAGACCCAATACGAGTG | 898bp | 100% | 0% | 0 |
| c43733.graph_c0-R | GATTTCCTCCAAGTAGC |  |  |  |  |
| c27804.graph_c0-F | TTTGGCTGTTATTGTTTGC | 288bp | 100% | 0% | 0 |
| c27804.graph_c0-R | CTCTTGGCCTTCTCCTCA |  |  |  |  |
| c34593.graph_c0-F | AACCATTCAACGCAACG | 406bp | 100% | 0% | 0 |
| c34593.graph_c0-R | CATTCTCCTTATCGGTCTT |  |  |  |  |
| c47921.graph_c0-F | TTCCATTCTGAGTTCCC | 564bp | 99% | 0% | 0 |
| c47921.graph_c0-R | GTATTCATTGCGATTTTGT |  |  |  |  |
| c21981.graph_c0-F | TATTCGCAGGCAAAGAT | 703bp | 100% | 0% | 0 |
| c21981.graph_c0-R | CAGCAAACAGCTAACAATG |  |  |  |  |

**Table S2.** Primers used to validate the mRNA -seq data for qRT-PCR

| the primers for qRT-PCR(5'-3') | | length | Identities | Gaps | E-value |
| --- | --- | --- | --- | --- | --- |
| *β-actin*F | TGTCGCCTTGGACTTTGAGC | 104bp | 100% | 0% | 0 |
| *β-actin*R | CATTGCCGATGGTGATAACT |  |  |  |  |
| c50430.graph_c0-F | CTCGGCACCTTCTTCCAATG | 100bp | 100% | 0% | 0 |
| c50430.graph_c0-R | ACGGCCAATCAAAATCACCA |  |  |  |  |
| c43733.graph_c0-F | GCTACCCGTTACTACACCGA | 96bp | 100% | 0% | 0 |
| c43733.graph_c0-R | CGTTTGCCATATCCCTGCTT |  |  |  |  |
| c27804.graph_c0-F | AATTGGGCAGCTTGATTGGT | 99bp | 100% | 0% | 0 |
| c27804.graph_c0-R | CATTGGCGGCATGGAAAGAT |  |  |  |  |
| c34593.graph_c0-F | CATTGTTGCCTGTGTCTCGG | 111bp | 100% | 0% | 0 |
| c34593.graph_c0-R | TTCATTTGCTCCTCGGTCAG |  |  |  |  |
| c47921.graph_c0-F | ACTAAACATTCGGGCTCCTCT | 121bp | 100% | 0% | 0 |
| c47921.graph_c0-R | TGTACTTGGGCGTATCGTGG |  |  |  |  |
| c21981.graph_c0-F | ACTGCCCGTCTACTTCAAGA | 108bp | 100% | 0% | 0 |
| c21981.graph_c0-R | CCTACAGCGGTGGTCTCTAC |  |  |  |  |

**Table S3.** Detailed information of topGO enrichment of the groups TC vs. TL (low temperature) and TC vs. TH (high temperature) and their sub-categories

| Groups | GO.ID | Term | Annotated | Significant | Expected | KS | Sub-categories |
| --- | --- | --- | --- | --- | --- | --- | --- |
| TC_vs_TL.topGO_BP | GO:0006412 | translation | 711 | 100 | 28.01 | 2.80E-23 | GO:0008152 metabolic process; |
| TC_vs_TL.topGO_BP | GO:0000022 | mitotic spindle elongation | 132 | 11 | 5.2 | 1.30E-14 | GO:0009987 cellular process;GO:0016043 cellular component organization |
| TC_vs_TL.topGO_BP | GO:0051298 | centrosome duplication | 83 | 3 | 3.27 | 6.50E-06 | GO:0009987 cellular process;GO:0016043 cellular component organization; |
| TC_vs_TL.topGO_BP | GO:0015986 | ATP synthesis coupled proton transport | 39 | 3 | 1.54 | 0.00023 | GO:0008152 metabolic process;GO:0009987 cellular process;GO:0051234 establishment of localization;GO:0051234 establishment of localization; |
| TC_vs_TL.topGO_BP | GO:0000028 | ribosomal small subunit assembly | 10 | 1 | 0.39 | 0.00029 | GO:0009987 cellular process; GO:0010926 anatomical structure formation;GO:0016043 cellular component organization; GO:0044085 cellular component biogenesis; |
| TC_vs_TL.topGO_BP | GO:0015031 | protein transport | 194 | 1 | 7.64 | 0.00186 | GO:0051179 localization;GO:0051234 establishment of localization; |
| TC_vs_TL.topGO_BP | GO:0006464 | cellular protein modification process | 642 | 14 | 25.29 | 0.00418 | GO:0008152 metabolic process;GO:0009987 cellular process |
| TC_vs_TL.topGO_BP | GO:0022900 | electron transport chain | 117 | 11 | 4.61 | 0.00616 | GO:0008152 metabolic process;GO:0009987 cellular process |
| TC_vs_TL.topGO_BP | GO:0007286 | spermatid development | 36 | 3 | 1.42 | 0.00637 | GO:0000003 reproduction;GO:0022414 reproductive process; GO:0009987 cellular process;GO:0032502 developmental process; |
| TC_vs_TL.topGO_BP | GO:0040009 | regulation of growth rate | 58 | 3 | 2.29 | 0.00811 | GO:0040007 growth; GO:0043473 pigmentation; GO:0065007 biological regulation |
| TC_vs_TL.topGO_BP | GO:0034660 | ncRNA metabolic process | 79 | 1 | 3.11 | 0.00858 | GO:0008152 metabolic process; GO:0009987 cellular process; |
| TC_vs_TL.topGO_CC | GO:0005840 | ribosome | 526 | 100 | 27.01 | 3.40E-15 | GO:0005623 cell;GO:0044464 cell part; |
| TC_vs_TL.topGO_CC | GO:0022625 | cytosolic large ribosomal subunit | 121 | 6 | 6.21 | 1.50E-12 | GO:0032991 macromolecular complex; |
| TC_vs_TL.topGO_CC | GO:0022627 | cytosolic small ribosomal subunit | 86 | 11 | 4.42 | 3.20E-10 | GO:0005623 cell;GO:0044464 cell part;GO:0032991 macromolecular complex;GO:0044422 organelle part; |
| TC_vs_TL.topGO_CC | GO:0044451 | nucleoplasm part | 109 | 1 | 5.6 | 0.0034 | GO:0005623 cell;GO:0044464 cell part;GO:0044422 organelle part |
| TC_vs_TL.topGO_CC | GO:0000228 | nuclear chromosome | 51 | 5 | 2.62 | 0.0046 | GO:0005623 cell;GO:0044464 cell part;GO:0043226 organelle;GO:0044422 organelle part |

| Groups | GO.ID | Term | Annotated | Significant | Expected | KS | Sub-categories |
| --- | --- | --- | --- | --- | --- | --- | --- |
| TC_vs_TL.topGO_CC | GO:0005622 | intracellular | 3204 | 201 | 164.5 | 0.006 | GO:0005623 cell;GO:0044464 cell part; |
| TC_vs_TL.topGO_CC | GO:0005753 | mitochondrial proton-transporting ATP sy... | 36 | 2 | 1.85 | 0.0082 | GO:0005623 cell;GO:0044464 cell part;GO:0031975 envelope;GO:0032991 macromolecular complex |
| TC_vs_TL.topGO_CC | GO:0005737 | cytoplasm | 2078 | 179 | 106.69 | 0.0089 | GO:0005623 cell;GO:0044464 cell part;GO:0043226 organelle;GO:0044422 organelle part |
| TC_vs_TL.topGO_CC | GO:0005861 | troponin complex | 7 | 3 | 0.36 | 0.0099 | GO:0005623 cell;GO:0044464 cell part;GO:0044421 extracellular region part |
| TC_vs_TL.topGO_MF | GO:0003735 | structural constituent of ribosome | 477 | 91 | 18.12 | < 1e-30 | GO:0005198 structural molecule activity; |
| TC_vs_TL.topGO_MF | GO:0003824 | catalytic activity | 4896 | 152 | 185.98 | 4.00E-06 | GO:0003824 catalytic activit |
| TC_vs_TL.topGO_MF | GO:0005525 | GTP binding | 199 | 8 | 7.56 | 2.70E-05 | GO:0005488 binding |
| TC_vs_TL.topGO_MF | GO:0042302 | structural constituent of cuticle | 49 | 9 | 1.86 | 6.90E-05 | GO:0005198 structural molecule activity |
| TC_vs_TL.topGO_MF | GO:0019843 | rRNA binding | 55 | 6 | 2.09 | 0.00087 | GO:0005488 binding |
| TC_vs_TL.topGO_MF | GO:0003924 | GTPase activity | 141 | 8 | 5.36 | 0.00257 | GO:0003824 catalytic activity |
| TC_vs_TL.topGO_MF | GO:0008168 | methyltransferase activity | 90 | 3 | 3.42 | 0.00325 | GO:0003824 catalytic activity |
| TC_vs_TL.topGO_MF | GO:0016757 | transferase activity, transferring glyco... | 127 | 1 | 4.82 | 0.00473 | GO:0016757 transferase activity, transferring glycosyl groups; |
| TC_vs_TL.topGO_MF | GO:0008553 | hydrogen-exporting ATPase activity, phos... | 34 | 2 | 1.29 | 0.00629 | GO:0003824 catalytic activity;GO:0005215 transporter activity; |
| TC_vs_TL.topGO_MF | GO:0030414 | peptidase inhibitor activity | 38 | 1 | 1.44 | 0.00727 | GO:0030234 enzyme regulator activity; |
| TC_vs_TL.topGO_MF | GO:0046933 | proton-transporting ATP synthase activit... | 27 | 3 | 1.03 | 0.00922 | GO:0005215 transporter activity |
| TC_vs_TL.topGO_MF | GO:0008565 | protein transporter activity | 33 | 1 | 1.25 | 0.00927 | GO:0005215 transporter activity |
| TC_vs_TL.topGO_MF | GO:0005388 | calcium-transporting ATPase activity | 22 | 7 | 0.84 | 0.00975 | GO:0005215 transporter activity;GO:0003824 catalytic activity |
| TC_vs_TH.topGO_BP | GO:0006412 | translation | 711 | 36 | 16.21 | 1.80E-17 | GO:0008152 metabolic process;GO:0009987 cellular process; |
| TC_vs_TH.topGO_BP | GO:0000022 | mitotic spindle elongation | 132 | 13 | 3.01 | 2.50E-14 | GO:0016043 cellular component organization;GO:0009987 cellular process; |

| Groups | GO.ID | Term | Annotated | Significant | Expected | KS | Sub-categories |
| --- | --- | --- | --- | --- | --- | --- | --- |
| TC_vs_TH.topGO_BP | GO:0051298 | centrosome duplication | 83 | 12 | 1.89 | 4.30E-07 | GO:0009987 cellular process; GO:0016043 cellular component organization; |
| TC_vs_TH.topGO_BP | GO:0009451 | RNA modification | 23 | 1 | 0.52 | 0.00099 | GO:0008152 metabolic process; GO:0009987 cellular process; |
| TC_vs_TH.topGO_BP | GO:0015031 | protein transport | 194 | 3 | 4.42 | 0.00116 | GO:0051179 localization;GO:0051234 establishment of localization; |
| TC_vs_TH.topGO_BP | GO:0043086 | negative regulation of catalytic activit... | 29 | 1 | 0.66 | 0.0021 | GO:0065007 biological regulation; |
| TC_vs_TH.topGO_BP | GO:0006464 | cellular protein modification process | 642 | 9 | 14.64 | 0.00647 | GO:0008152 metabolic process; |
| TC_vs_TH.topGO_BP | GO:0007264 | small GTPase mediated signal transductio... | 187 | 2 | 4.26 | 0.00781 | GO:0009987 cellular process;GO:0043473 pigmentation;GO:0065007 biological regulation; |
| TC_vs_TH.topGO_BP | GO:0034470 | ncRNA processing | 42 | 2 | 0.96 | 0.00791 | GO:0008152 metabolic process;GO:0009987 cellular process; |
| TC_vs_TH.topGO_BP | GO:0042254 | ribosome biogenesis | 66 | 3 | 1.5 | 0.00868 | GO:0009987 cellular process;GO:0044085 cellular component biogenesis; |
| TC_vs_TH.topGO_BP | GO:0000280 | nuclear division | 68 | 2 | 1.55 | 0.00932 | GO:0009987 cellular process; GO:0016043 cellular component organization; |
| TC_vs_TH.topGO_CC | GO:0022627 | cytosolic small ribosomal subunit | 86 | 12 | 1.99 | 1.00E-12 | GO:0005623 cell;GO:0044464 cell part;GO:0032991 macromolecular complex;GO:0043226 organelle;GO:0044422 organelle part; |
| TC_vs_TH.topGO_CC | GO:0022625 | cytosolic large ribosomal subunit | 121 | 14 | 2.8 | 3.00E-12 | GO:0005623 cell;GO:0044464 cell part;GO:0032991 macromolecular complex; GO:0043226 organelle;GO:0044422 organelle part; |
| TC_vs_TH.topGO_CC | GO:0005840 | ribosome | 526 | 33 | 12.19 | 2.20E-07 | GO:0005623 cell;GO:0044464 cell part; GO:0032991 macromolecular complex; |
| TC_vs_TH.topGO_CC | GO:0044451 | nucleoplasm part | 109 | 4 | 2.53 | 0.0015 | GO:0005623 cell;GO:0044464 cell part;GO:0031974 membrane-enclosed lumen;GO:0043226 organelle;GO:0044422 organelle part; |
| TC_vs_TH.topGO_CC | GO:0005665 | DNA-directed RNA polymerase II, core com... | 13 | 1 | 0.3 | 0.0058 | GO:0005623 cell;GO:0044464 cell part;GO:0031974 membrane-enclosed lumen;GO:0032991 macromolecular complex;GO:0043226 organelle;GO:0044422 organelle part; |
| TC_vs_TH.topGO_CC | GO:0005730 | nucleolus | 48 | 3 | 1.11 | 0.006 | GO:0005623 cell;GO:0044464 cell part;GO:0031974 membrane-enclosed lumen;GO:0043226 organelle;GO:0044422 organelle part; |
| TC_vs_TH.topGO_CC | GO:0000228 | nuclear chromosome | 51 | 3 | 1.18 | 0.0066 | GO:0005623 cell;GO:0044464 cell part;GO:0043226 organelle;GO:0044422 organelle part; |

| Groups | GO.ID | Term | Annotated | Significant | Expected | KS | Sub-categories |
| --- | --- | --- | --- | --- | --- | --- | --- |
| TC_vs_TH.topGO_CC | GO:0044424 | intracellular part | 3108 | 80 | 72.04 | 0.007 | GO:0005623 cell;GO:0044464 cell part; |
| TC_vs_TH.topGO_CC | GO:0005761 | mitochondrial ribosome | 30 | 1 | 0.7 | 0.0092 | GO:0005623 cell;GO:0044464 cell part;GO:0031974 membrane-enclosed lumen;GO:0032991 macromolecular complex; GO:0043226 organelle;GO:0044422 organelle part; |
| TC_vs_TH.topGO_MF | GO:0003735 | structural constituent of ribosome | 477 | 32 | 11.43 | 2.00E-27 | GO:0005198 structural molecule activity; |
| TC_vs_TH.topGO_MF | GO:0003824 | catalytic activity | 4896 | 101 | 117.34 | 7.50E-07 | GO:0003824 catalytic activity; |
| TC_vs_TH.topGO_MF | GO:0042302 | structural constituent of cuticle | 49 | 11 | 1.17 | 5.40E-05 | GO:0005198 structural molecule activity; |
| TC_vs_TH.topGO_MF | GO:0005525 | GTP binding | 199 | 4 | 4.77 | 9.70E-05 | GO:0005488 binding; |
| TC_vs_TH.topGO_MF | GO:0004767 | sphingomyelin phosphodiesterase activity | 28 | 1 | 0.67 | 0.00017 | GO:0003824 catalytic activity |
| TC_vs_TH.topGO_MF | GO:0008168 | methyltransferase activity | 90 | 2 | 2.16 | 0.00241 | GO:0003824 catalytic activity; |
| TC_vs_TH.topGO_MF | GO:0016757 | transferase activity, transferring glyco... | 127 | 1 | 3.04 | 0.00294 | GO:0003824 catalytic activity; |
| TC_vs_TH.topGO_MF | GO:0019843 | rRNA binding | 55 | 4 | 1.32 | 0.00514 | GO:0005488 binding; |
| TC_vs_TH.topGO_MF | GO:0030414 | peptidase inhibitor activity | 38 | 2 | 0.91 | 0.00564 | GO:0030234 enzyme regulator activity; |
| TC_vs_TH.topGO_MF | GO:0003924 | GTPase activity | 141 | 3 | 3.38 | 0.00929 | GO:0003824 catalytic activity; |
